# Preschool Minority Children’s Persian Vocabulary Development: A Language Sample Analysis

**DOI:** 10.3389/fpsyg.2022.761228

**Published:** 2022-03-17

**Authors:** Mohamad Reza Farangi, Saeed Mehrpour

**Affiliations:** Department of Language and Linguistics, Shiraz University, Shiraz, Iran

**Keywords:** children’s language, vocabulary development, background TV, socioeconomic status, early language development

## Abstract

This study linked background TV and socioeconomic status (SES) to minority children’s Persian vocabulary development. To this end, 80 Iranian preschool children (aged 5–6 years old) from two minority groups of Arabs and Turks were selected using stratified random sampling. They were simultaneous bilinguals, i.e., their mother tongue was either Arabic or Azari and their first language was Persian. Language sample analysis (LSA) was used to measure vocabulary development through a 15-min interview by language experts (PhD in applied linguistics). The LSA measures included total number of utterances (TNU), total number of words (TNW), total number of new words (NDW), and mean length of utterance (MLU). A series of independent-samples *t* test, paired-samples *t* test, and repeated measures MANOVA tests were ran to examine data. Results showed significant improvements in children’s vocabulary scores from pretest to posttest for all children. In addition, high-SES children scored higher on the vocabulary measures in pretest and posttest. Moreover, background TV was associated with higher means in the TNW and the NDW in groups. The researchers concluded that background TV may be related to higher vocabulary scores in low-SES families as it may compensate for some linguistics gaps in these families including lower amount of child-parent interaction, play, and parents’ level of literacy.

## Introduction

Minority children’s language development in mainstream languages has often been associated with some difficulties (Hardy and Jurecka, 2018). Research has shown that minority students are able to overcome hurdles in their path to academic achievement only when individual, cognitive, linguistic, and SES factors are considered (Maluch et al., 2015; Hopp et al., 2019). Accordingly, this study was an attempt to examine how SES mediated by the presence of background TV can influence vocabulary development among Iranian minority children.

### Language and Socioeconomic Status

Socioeconomic status (SES) is described by one or an amalgamation of these measures: (a) family income; (b) family education; and (c) occupation (Vanormelingen and Gillis, 2016). Research on SES is mixed with majority of studies arguing for moderate to strong effects on children’s language development. In a very recent review of the literature, SES and educational achievement were associated, and Broer et al. (2019) argued that narrowing SES gap in educational domain has been of interest to researchers for decades. As determinants of SES, parental education and family income are believed to be associated with faster growth in children’s vocabulary development (Hoff, 2003). Mothers of high-SES families talk not only more but also differently to their children compared to their low-SES counterparts (Andonova, 2015; Vanormelingen and Gillis, 2016). Fewer social problems and more prosocial behavior (Letourneau et al., 2013), higher participation in schools (Veland et al., 2015), better grades (Lekholm and Cliffordson, 2008), and outperforming others in achievement measures (Sirin, 2005) have been associated with a high-SES background. Hart and Risley (1995) found that children from low-SES families were exposed to 616 words per hour on average, as compared to middle-SES children who received 1,251 words per hour and high-SES children who received 2,153 words per hour. These results may be outdated but still emphasizes the importance of SES on children’s language development.

Hoff (2003) recruited 33 high-SES and 30 middle-SES families to explore SES effects on early vocabulary development. In this study, children from high-SES families showed a larger vocabulary repertoire compared to their middle-SES counterparts. Sirin (2005) conducted a meta-analysis to examine SES effects on learners’ academic achievement. A moderate to strong relationship between SES and students’ achievement was observed, and SES effects were moderated by the learners’ grade level, minority status, and school location. Andonova (2015) investigated associations between age, gender, and maternal education in communicative development of 153 Bulgarian toddlers. It was found that children of mothers with higher education produced more words than children of mothers with a high school diploma. Vanormelingen and Gillis (2016) showed that low-SES indigenous Dutch speaking caregivers speak less and respond less informatively to their children compared to their high-SES counterparts.

Eilertsen et al. (2016) argued that children’s cognitive performance was associated with their family SES and parental education was an important predictor while their income did not show a strong effect. These results may indicate that the literature on SES is mixed and more studies are needed to expand our understanding of the topic. Gathercole et al. (2016) study on 732 Welsh and English learners age 3 to over 60 from different SES groups showed that SES had a strong effect on the children’s performance on vocabulary and grammar measures. In a recent study conducted by DeVries et al. (2018), students’ achievement was predicted by family SES level as better grades in math and reading were directly related to parental education. Kyriakides et al. (2019) examined the effects of SES gap on students’ achievement in mathematics and language courses and showed that effective teaching techniques and classroom practices can fill SES gap. These results also highlighted the importance of mediators on SES roles in a given society. Furthermore, Torres (2018) considered family SES as a source of inequality and argued that teachers can be strong moderators in this regard as their results showed a higher proportion of effective teachers in classrooms with students from low-SES backgrounds. Majority of studies on SES showed that it was moderated by several variables. One of these variables which are considered in this study is background TV exposure.

### Language and Background TV

TV has been directly associated with family SES (Zimmerman et al., 2009; American Academy of Pediatrics, Council on Communications and Media, 2011). Despite prevalence of different media, TV is continued to be the main entertaining and educational source for children. How it affects language development depends on quantity (hours of exposure) and quality (educational, entertaining, etc.) of exposure. Exposure to TV is categorized as foreground and background. Background TV is defined as having a program which is not of interest to a child or when the TV is left on with no one watching it (Masur et al., 2016). Young children in the United States are exposed to foreground exposure about one to one and half an hour on average (Rideout, 2013 as cited in Masur et al., 2016, p.12) plus an average of 5.5 h of background exposure on a daily basis (Lapierre et al., 2012). Research on exposure to TV has provided somehow mixing results (Christakis et al., 2009; Pempek et al., 2014; Masur et al., 2016; Madigan et al., 2020). Background TV has been associated with children’s attentional processes (Setliff and Courage, 2011), their vocalization (Christakis et al., 2009), parental involvement (Kirkorian et al., 2009), and quantity and quality of interactions both in positive and negative ways (Madigan et al., 2020). It is recently differentiated from the foreground TV in research; as a result, the literature is not only scarce but also limited to some specific countries, such as the United States. So, the researchers in this study felt the need to expand this line of research to other parts of the world. Results of some studies are reviewed below.

Anderson and Pempek (2005) associated TV viewing with very young children’s language growth. Kamila et al. (2007) interviewed a large number of families with children of 30–33 months and 5.5 years old and argued that background exposure was associated with behavioral problems and fewer social skills. According to Kirkorian et al. (2009), for children of 12-, 24-, and 36-month-old, parent-child interactions (their play in this study) reduced in the presence of a background TV at home. Similarly, Christakis et al. (2009) conducted a research on 329 2–48-month-old children and showed a significant reduction in children’s vocalization and adult’s word counts as a result of background exposure. Two-phase study of Zimmerman et al. (2009) on 275 children age 2–48 months showed that screen time (both foreground and background) had a negative relationship with their language development.

However, when talking about children’s language, we should consider both quantity and quality of their language. Pempek et al. (2014) compared quantity and quality of parent-child interactions with and without background TV exposure. Accordingly, number of words and utterance per minutes as measures of quantity and number of new words (NDW) and length of utterances as measures of quality showed reduction while syntactic complexity (i.e., length of utterances) was untouched. In a similar vein, Masur et al. (2016) investigated 25 American mother’s interactions with their children age 13–17 months and argued that their expressive vocabulary reduced due to background exposure and both quantity and quality of maternal speech were affected. However, these results should be taken into account cautiously as many factors affect children’s language growth. For example, sensitive parenting behavior (Funder and Ozer, 2019), preterm birth (Madigan et al., 2015), number of words spoken by caregivers (Wade et al., 2018), and SES (Hart and Risley, 1995) were found to largely predict children’s language development. Hence, background exposure is an element predicting a multifactorial construct (i.e., children’s language development). So, further studies in other contexts are necessary to validate or reject these results.

### Present Study

The present study extends the line of research on SES and background TV with following gaps in the mind of researchers. First, these variables have been largely ignored in non-Anglo-Saxon countries (Vanormelingen and Gillis, 2016). Iran, as the context of this study, hosts the first round of these types of research to the best of researchers’ knowledge. Furthermore, while majority of previous studies focused on toddlers, the present study focused on preschool children (aged 5–6 in Iran). Then, this study examined groups of children whose first language was Farsi (Persian) while they used their mother tongues (Kurdish and Azari) at home. Farsi is an under-researched language with a scarce published literature in this regard. Exploring how different languages can grow together can provide us with a more comprehensive picture of children’s language development profile. For this purpose, this study attempted to investigate how SES and background TV exposure were associated with vocabulary development among children.

## Materials and Methods

### Participants

From a population of 562 children, 80 male and female children ages 5–6 years old (*M* = 5.9, *SD* = 0.74) from local preschools in Ahvaz and Tabriz, south west and north west of Iran participated in the present study. Ahvaz and Tabriz are multi-cultural and multi-lingual cities in Iran populated by Persian, Azari, and Arabic speaking residents. Based on a preliminary analysis, the participants were able to understand and speak Persian fluently. Their mother tongues were Arabic and Azari. In this study, mother tongue was defined as the language spoken at home. Stratified random sampling was used in a way that, first, the cities were divided into two strata based on SES map of municipalities. Families were selected from these two SES strata. Forty families belonged to high-SES (HSE) category and 40 to low-SES (LSE) one. Based on the literature, criteria for SES were mother’s educational degree and family income assessed indirectly through kindergarten locations in order to avoid false reports. Kindergartens in low-SES neighborhoods are predominantly situated in old houses with two or three rooms. Boys and girls spend 4 h a day, 5 days a week there. Popular activities in these centers are playing with toys, watching animated cartoons, playing soccer (for the boys), painting, Quran recitation, making handicrafts, etc. Their tuition fee is normally low. Kindergartens in high-SES neighborhoods are usually located in big houses with lots of furniture, which follow a systematic curriculum. They use critical thinking activities, puzzles, robotics, yoga, sport activities, and some limited English classes. The tuition fee in these centers is very high.

In the high-SES group, mothers had a university degree (BA = 25, MA = 10, and PhD = 5) and in the low-SES group, they had mainly completed high school education (*N* = 31) or had a diploma (*N* = 9). Twenty-one mothers (52%) in the high-SES group reported to have a full-time job. Eighteen mothers (46%) in both groups worked part-time out of the house. All fathers reported to have a full-time job outside the house. In the high-SES group, 13 kids were an only child, 10 girls were the first child, five boys were the last child, and others were middle children; in the low-SES group, eight boys were the first child, 14 girls were the first child, three kids were an only child, and others were middle children. Both parents and children filled consent form and agreed to participate in the study. The researchers promised to follow anonymity principles throughout the research period.

### Materials and Instruments

#### Language Sample Analysis

Language sample analysis (LSA) measure was used to collect data as described by Hoff and Naigles (2002). It is an important instrument for collecting samples of children’s speech (Paul and Norbury, 2012). Several studies used this tool to assess quantity and quality indicators in children’s language (e.g., Horton-Ikard, 2010; Oetting et al., 2010; Miller et al., 2011; Paul and Norbury, 2012; Stockman et al., 2013; Westerveld and Claessen, 2014). Miller et al. (2011) recommended 10–15-min language samples (50–100 utterances) as a reliable sample size. This measure has the following components: total number of utterances (TNU; i.e., mean number of utterances; independent clauses) produced by the test takers per minute, total number of words (TNW; i.e., frequency of each and every word produced per minute), NDW (i.e., total number of novel words in which different forms of the same root were treated as the same word and were not counted. Thus, “go, went, and gone” were counted as one word as were “apple and apples”), and mean length of utterance (MLU; i.e., syntactic complexity considered as an index of richness of linguistic environment).

The interviews included simple questions of routine activities (e.g., what did you eat for dinner?/ what is your favorite toy?/what is your favorite play?/etc.). Systematic Analysis of Language Transcripts (SALT; Miller and Iglesias, 2010) principles were used for transcriptions of language samples. A minute by minute marking method was used for transcription. All transcripts were examined to remove inconsistencies. Those utterances which could not be heard were marked as unintelligible. On the basis of these transcripts, the number of words produced per minute, the number of utterances produced per minute, the number of new words produced per minute, and the MLUs were considered as quantitative and qualitative measures of vocabulary size. Ratings were done by two trained raters. In order to check interrater reliability, the researchers used Cohen’s Kappa Statistics. For this purpose, all the transcripts were rated by the raters and the calculated Kappa statistics was (0.85) indicating near perfect agreement between the raters.

#### Media Exposure Profile

In order to verify hours of children’s exposure to background TV, the researchers devoted a media exposure profile to each child, which was completed by one of his/her family members every day. Exposure profiles were collected on the weekends through WhatsApp. The profile included following items in which the first two items determined hours of exposure to background TV.

- How many hours did the child watch TV?
- How many hours was the TV on at home?
- How many hours did the child spend on some other devices such as smartphones and tablets?
- What were main TV programs broadcasted?
- How many hours did the child spend playing with a family member or a friend?

### Procedure

For statistical analyses, four groups of 20 participants were constructed based on SES and background exposure. As the researchers in this study could not find a single study recording children’s hours of exposure to background TV in Iran, we used mean hours reported in foreign studies. Surveying prevalence of background exposure in US homes, Lapierre et al. (2012) found that poor families were normally exposed to almost 6 h of background TV. For the purpose of this study, we considered 8 h for high background TV exposure and 4 h for low background TV. From among 562 families who agreed to participate, only 80 families could finish the study and their data were used. Descriptions of the groups are provided below:

#### High Socioeconomic Status-High Background TV (HSE_HB)

This group comprised of nine girls and 11 boys. The media profile showed that TV was on for a mean of 8 h a day when the children were at home. These children had registered in kindergartens located in rich regions of their cities and their mothers had a university degree.

#### High Socioeconomic-Low Background TV

Eight female and 12 male children were included in this group. Based on the media profile reports, TV was on for a mean of 4 h when the children were at home. Children in this group had registered in kindergartens located in rich areas of their cities and their mothers had a university degree.

#### Low Socioeconomic-High Background TV

Seven female and 13 male children built up the third group. According to profile reports, TV was on for a mean of 8 h when the children were at home. Participants in this group had registered in kindergartens located in poor areas of their cities and their mothers did not have a university degree.

#### Low Socioeconomic-Low Background TV

This group comprised of 10 female and 10 male children and TV was on for a mean of 4 h a day during children’s presence. Members of this group had registered in kindergartens located in poor areas of their cities and their mothers did not have a university degree.

During the study period, kindergartens were closed and children spent most of their time at home. They took pretest 1 week before commencement of study and posttest 1 week after the end of it. Independent-samples *t* test, paired-samples *t* test, and repeated measures MANOVA tests were used in the SPSS software version 21 to analyze data and interpret results.

### A Description of Iranian TV Channels

Iranian TV channels are organized and controlled by the Broadcasting Organization. There are four national channels (called channels 1–4) plus one provincial or local channel for each province throughout the country. All the channels broadcast a variety of programs, such as news, documentaries, movies, and children programs (e.g., cartoons). In addition to the above channels, there are about 40 regional TV channels in Iran. These digital channels can be accessed only by those who own a digital channel receiver or a modern TV with a built-in digital receiver technology. These digital stations present a host of specialized programs, such as Quran, Education, Documentary, Health, News, and Child & Adolescent. Language of all national channels is Persian and provincial channels produce content in regional languages.

## Results and Discussion

Descriptive and inferential statistics are presented in this section. This study associated background exposure and family SES to children’s Persian vocabulary development. Table 1 reports descriptive statistics on the background exposure.

**Table 1:** Exposure to background TV.

Groups	Mean	Range
HSE_HB	7 h/41 min	6–9 h
HSE_LB	4 h/18 min	4–5 h
LSE_HB	7 h/23 min	6–10 h
LSE_LB	4 h/22 min	4–5 h

Mean and range of daily background TV were provided in Table 1. Table 2 provided descriptive statistics on the measures of vocabulary development.

**Table 2:** Descriptive statistics for Language sample analysis (LSA) measures.

	Number of utterances	Number of new words	Number of word types	Mean length of utterance
Vocabulary measure	897	1,365	625	*M* = 3.12 *SD* = 0.87

Following that, SES effects on the vocabulary development were investigated. Means and SDs for SES are provided in Table 3.

**Table 3:** Descriptive statistics for the socioeconomic status (SES).

Groups	TNU		TNW		NDW		MLU	
	Pre	Post	Pre	Post	Pre	Post	Pre	Post
High-SES	M: 3.99	M: 5.43	M: 14.33	M: 21.44	M: 1.88	M: 2.66	M: 2.42	M: 3.11
	SD: 0.26	SD: 0.88	SD: 1.87	SD: 2.24	SD: 0.66	SD: 0.74	SD: 0.47	SD: 0.56
Low-SES	M: 3.77	M: 4.21	M: 13.89	M: 18.56	M: 1.45	M: 2.01	M: 2.01	M: 2.64
	SD: 0.75	SD: 1.01	SD: 2.44	SD: 1.68	SD: 0.64	SD: 0.74	SD: 0.23	SD: 0.54

As seen, between-group differences were observed in the pretest and posttest. Independent-samples *t* test was used to investigate differences between the high and low-SES groups on the language measures. Results showed significant differences between the means of TNU [*t* (78) = 2.244, *p* < 0.001], TNW [*t* (78) = 1.985, *p* < 0.001], NDW [*t* (78) = 2.152, *p* < 0.001], and MLU [*t* (78) = 2.052, *p* < 0.001] in the pretest. These results indicated that SES had its effects prior to the onset of study. Regarding the posttest, similarly, results of independent-samples *t* test showed statistically significant differences between the high- and low-SES groups {TNU [*t* (78) = 1.853, *p* < 0.001], TNW [*t* (78) = 1.965, *p* < 0.001], NDW [*t* (78) = 1.754, *p* < 0.001], and MLU [*t* (78) = 1.684, *p* < 0.001]} indicating that SES effects were observed in the posttest too.

Moreover, paired-samples *t* test was used to investigate differences between pretest and posttest means of the high- and low-SES groups. Results for the high-SES group showed that these differences were statistically significant {TNU [*t* (79) = −7.365, *p* < 0.001], TNW [*t* (79) = −7.263, *p* < 0.001], NDW [*t* (79) = −7.668, *p* < 0.001], and MLU [*t* (79) = −7.852, *p* < 0.001]}. Similarly, in the low-SES group, differences in the pretest and posttest means were statistically significant {TNU [*t* (79) = −8.675, *p* < 0.001], TNW [*t* (79) = −8.478, *p* < 0.001], NDW [*t* (79) = −8.679, *p* < 0.001], and MLU [*t* (79) = −8.975, *p* < 0.001]}.

According to these results, children in the high-SES group had higher means in the pretest and posttest compared to the children in the low-SES group. They produced more utterances per minute, more words per minute, more new words per minute, and their MLUs was higher. These results were consistent with results of other studies. Sirin (2005), in an extensive review of literature, showed that there was a moderate to strong relationship between SES and students achievement. Hagedoorn et al. (2016) showed that low-SES indigenous Dutch speaking caregivers speak less and respond less informatively to their children compared to their high-SES counterparts. Eilertsen et al. (2016) highlighted SES effects on children’s cognitive performance in the Scandinavian area. Gathercole et al. (2016) showed that SES had a strong effect on children’s performance on these abilities. However, DeVries et al. (2017, 2018) argued that students whose parents had lower academic degrees did not perform less successfully in their academic achievement, which were not consistent with our results. Differences in these results may arise from the fact that DeVriess’ studies measured children’s comprehension abilities while the current study measured children’s production abilities.

Then, the effects of background exposure on children’s vocabulary growth were measured. Descriptive statistics are provided in Table 4.

**Table 4:** Descriptive statistics for background TV.

Group	TNU		TNW		NDW		MLU	
	Pre	Post	Pre	Post	Pre	Post	Pre	Post
High TV	M: 3.78	M: 4.46	M: 15.28	M: 23.54	M: 2.76	M: 3.98	M: 2.51	M: 3.13
	SD: 0.46	SD: 0.78	SD: 3.38	SD: 4.23	SD: 0.23	SD:0.87	SD: 0.57	SD: 0.14
Low TV	M: 3.79	M: 4.39	M: 15.14	M: 20.63	M: 2.89	M: 3.43	M: 2.56	M: 3.01
	SD: 0.36	SD: 0.54	SD: 3.33	SD: 3.11	SD: 0.36	SD: 0.64	SD: 0.13	SD: 0.12

As shown in Table 4, between-group differences on the vocabulary measures in the pretest and posttest can be observed.

Similar to analyses for SES, independent-samples *t* test was used to investigate differences between the high and low background groups on the language measures. Results showed that means of TNU [*t* (78) = −3.265, *p* > 0.001], TNW [*t* (78) = −3.120, *p* > 0.001], NDW [*t* (78) = −2.874, *p* > 0.001], and MLU [*t* (78) = −2.652, *p* > 0.001] did not show a statistically significant difference in the pretest. It indicated that there were no between-group differences regarding the background exposure in the pretest. Independent-samples *t* test results for the posttest showed statistically significant differences between the high and low background groups in TNW [*t* (78) = 1.123, *p* < 0.001] and NDW [*t* (78) = 1.110, *p* < 0.001] measures but not in TNU [*t* (78) = −1.232, *p* > 0.001], and MLU [*t* (78) = −1.168, *p* > 0.001] measures. Accordingly, children had significantly higher means in production of words per minute and new words per minute in the posttest while differences between the total number of utterance and MLU were not statistically significant. According to these results, it seems that the background exposure has been associated with higher use of words per minute and new words per minute by children. The TNU and the MLUs did not show significant differences in this study. These results were similar and different from the result of Pempek et al. (2014) as they showed a reduction in the number of words, number of utterances, and number of new words (differences) but no change in the length of utterances (similarity).

Moreover, the researchers used paired-samples *t* test to investigate between-group differences in the pretest and posttest. The high background group showed significant differences in their pretest and posttest means {TNU [*t* (79) = −9.345, *p* < 0.001], TNW [*t* (79) = −8.875, *p* < 0.001], NDW [*t* (79) = −9.456, *p* < 0.001], and MLU [*t* (79) = −8.286, *p* < 0.001]}. Moreover, between-group differences in the pretest and posttest means for the low background group were statistically significant {TNU [*t* (79) = −6.452, *p* < 0.001], TNW [*t* (79) = −7.356, *p* < 0.001], NDW [*t* (79) = −8.214, *p* < 0.001], and MLU [*t* (79) = −8.235, *p* < 0.001]}. Accordingly, children’s vocabulary scores improved from the pretest to the posttest in the low and high background groups.

These results supported some studies and contradicted some others. Pempek et al. (2014) showed that the background exposure reduced words and utterance per minutes and number of new words in parent-child discourse which is in contrast with the present study results. This difference may arise from different contexts in which these studies were conducted and the role of background TV in these contexts. Anderson and Pempek (2005) associated TV viewing with very young children’s language growth which is in line with the present study results. According to Kirkorian et al. (2009), parent-child interactions (i.e., play) varied by the background exposure. Zimmerman et al. (2009) showed that screen time (both foreground and background) had a negative association with children’s language development. Masur et al. (2016) showed that expressive vocabulary of children got lower due to the background exposure and quantity and quality of maternal speech were affected. However, Madigan et al. (2020), in their systematic review of screen time and language skills, warned researchers about too early conclusions in this regard. The researchers in this study, in line with the warnings put forward by Madigan et al. (2020), believed that in some contexts, the background exposure may improve some aspects of children’s vocabulary development.

Finally, how SES effects were mediated by the background exposure was also considered. For this purpose, based on level of SES and rate of exposure, four groups were formed. Descriptive statistics are provided in Table 5.

**Table 5:** The interaction effects of SES and background TV.

Group	TNU		TNW		NDW		MLU	
	Pre	Post	Pre	Post	Pre	Post	Pre	Post
HSE_HB	M: 4.23	M: 6.25	M: 17.26	M: 22.36	M: 2.36	M: 4.38	M: 2.89	M: 3.88
	SD: 0.56	SD: 0.78	SD: 2.58	SD: 3.24	SD: 0.23	SD: 0.57	SD: 0.77	SD: 1.11
HSE_LB	M: 4.89	M: 5.25	M: 16.89	M: 19.25	M: 4.12	M: 2.89	M: 2.46	M: 3.12
	SD: 0.45	SD: 1.17	SD: 3.69	SD: 2.57	SD: 0.36	SD: 0.74	SD: 0.23	SD: 0.28
LSE_HB	M: 4.26	M: 5.75	M: 15.65	M: 19.56	M: 2.78	M: 4.14	M: 2.22	M: 3.62
	SD: 0.22	SD: 1.10	SD: 2.35	SD: 3.47	SD: 0.10	SD: 0.55	SD: 0.33	SD: 1.42
LSE_LB	M: 3.99	M: 4.87	M: 15.23	M: 18.52	M: 2.12	M: 4.15	M: 2.18	M: 2.78
	SD: 0.11	SD: 0.54	SD: 3.21	SD: 2.87	SD: 0.19	SD: 0.41	SD: 0.41	SD: 0.38

In order to investigate between-group differences, one-way repeated measures MANOVA statistics was used. In this model, continuous dependent variables were TNU means, TNW means, NDW means, and MLU means measured over two time points (i.e., pretest and posttest). Independent variables in this model were High Socioeconomic Status-High Background TV (HSE_HB), High Socioeconomic-Low Background TV (HSE_LB), Low Socioeconomic-High Background TV (LSE_HB), and Low Socioeconomic-Low Background TV (LSE_LB).

For TNU, Wilks lambda results showed significant between-group differences in the pretest and posttest [*F* (6, 150) = 4.235, *p* < 0.05]. Accordingly, there were significant differences in combined dependent variables among groups. However, as one-way repeated measure MANOVA is an omnibus test, it does not tell us where between-group differences lay. For this purpose, Bonferroni *post-hoc* test was used and its results showed significant differences between HSE-HB and LSE-LB with other groups while differences between HSE-LB and LSE-HB were not statistically significant (*p* < 0.05).

Considering TNW, Wilks lambda results showed significant between-group differences in the pretest and posttest [*F* (6, 150) = 5.783, *p* < 0.05]. According to Table 5, HSE_HB had the highest mean and LSE-LB had the lowest mean in the posttest. Bonferroni *post-hoc* test results showed significant differences between HSE-HB and LSE-LB with other groups while differences between HSE_LB and LSE_HB were not statistically significant (*p* < 0.05).

For NDW, Wilks lambda results showed significant between-group differences in the pretest and posttest [*F* (6, 150) = 6.332, *p* < 0.05]. This indicated significant differences in combined dependent variables among groups. Table 5 statistics showed that HSE_HB had the highest mean among groups. Results of Bonferroni *post-hoc* test showed significant differences between HSE-HB with other groups while differences between HSE_LB, LSE_HB, and LSE_LB were not statistically significant (*p* < 0.05).

Finally, repeated measures MANOVA was ran for MLU. Results of Wilks Lambda showed significant between-group differences in the pretest and posttest [*F* (6, 150) = 4.872, *p* < 0.05] indicating significant differences in combined dependent variables among groups. Table 5 showed that HSE_HB had the highest mean and LSE_LB had the lowest mean among groups. Bonferroni *post-hoc* test showed significant differences between the groups in the posttest (*p* < 0.05).

Based on these statistical results, it can be argued that, probably, children in the high-SES group who were exposed to the high background TV scored higher than the other groups in their vocabulary development measures. Moreover, children in the low-SES group who were exposed to the high background TV may have shown improvement in their vocabulary measures.

## Conclusion

This study focused on filling the gaps in the literature on children’s vocabulary development. The context was an Asian under-researched area (i.e., Iran) and the literature on the language (i.e., Persian) was scarce. While the researchers could find a few studies on SES of Iranian families, to the best of our knowledge, this was the first study investigating the effects of background TV in this context. To make the point clearer, most of the Iranian families were not acquainted with the concept of background TV and could not differentiate it from foreground TV.

Regarding the results, all children in this study demonstrated improvement in their vocabulary repertoire during the study. These results were in line with the well-established literature on children’s vocabulary development according to which development will continue over time regardless of striking differences in contexts (Pempek et al., 2014). Besides, the results showed that children in the high-SES groups had higher means both in the pretest and posttest compared to the low-SES groups, which can be explained by fewer hours of fathers’ presence at home in the low-SES families, lower linguistic abilities of parents in these families, and higher population of these families (Leech et al., 2013). At the same time, exposure to the high background TV was significantly associated with children’s vocabulary development as the results showed that vocabulary development rate was higher in the high background groups. Based on these results, the researchers argued that the background TV may be associated with children’s vocabulary development.

Moreover, high background TV was linked to production of higher number of words per minute and higher number of new words per minute in the posttests but not in number of utterances per minute and MLU. This was an interesting result as previous research has mostly argued that the background TV was negatively associated with children’s language development (Christakis et al., 2009; Zimmerman et al., 2009). In addition, when SES effects were mediated by the background TV, interesting results emerged. Accordingly, the high background TV was related to higher vocabulary development among children in the low-SES families. It can be argued that, while children in the high-SES families may have experienced more favorable conditions in their linguistic development, in the low-SES families, the background exposure could be an important mediator in improving their vocabulary development as it may compensate for weaker children-parent interactions and play. These results may be enforced, specially, in the pandemic era, when children have to spend most of their time at home and have less access to their friends outside. During this time, TV may turn to a very important medium available to children through which they can entertain themselves and even learn new things. In the last 2 years of COVID-19 pandemic, Iranian kindergartens and schools were closed and the Islamic Republic of Iran Broadcasting organization devoted a channel for training and education of students in the country. Therefore, research on the screen time should be updated considering these new conditions around the world.

## Implications

These results may have several implications for children’s language development research. First of all, in developing countries like Iran, where majority of families come from a low-SES background, children’s language development in all its different aspects should be taken into account. This is a very neglected area which necessitates more attention from the stakeholders. Furthermore, maternal education should be considered as an important socioeconomic factor and planned for. According to statistics provided by the Sadreghazi (2015), the number of educated women has increased significantly in Iran in the last 2 decades. However, based on Iran rank in the world, the government should provide more educational opportunities for Iranian women. Mothers with higher level of education can enlighten and affect whole family. At the same time, families need to be educated about detrimental and beneficial effects of background TV on their children’s language development. For low-SES families, language development programs which correspond to families’ financial background should be designed to improve quality of background TV exposure at home and help children in their vocabulary development.

## Limitations and Suggestions for Future Studies

This study had its own limitations. Although it is very difficult to collect data when working with children, larger sample sizes can provide researchers and readers with more comprehensive results. There is a need for more longitudinal studies to investigate how children’s vocabulary development changes over a longer period of time. Moreover, different measures of vocabulary development can be used to enrich data analysis. Besides, an interventional design is needed to control effects of some intervening variables including foreground TV and other electronic devices (e.g., smartphones and tablets) which were untouched in this study. The correlational nature of this study may have limited reporting the results with strong words. Furthermore, lack of data on the quality of background TV content and the self-report structure of measuring background TV were other limitations of this study.

## Data Availability Statement

The datasets presented in this article are not readily available because permission was not given to publish the datasets. Requests to access the datasets should be directed to the corresponding author.

## Ethics Statement

The studies involving human participants were reviewed and approved by Ethics committee at Shiraz University. Written informed consent to participate in this study was provided by the participants’ legal guardian/next of kin.

## Author Contributions

All authors listed have made a substantial, direct, and intellectual contribution to the work and approved it for publication.

## Conflict of Interest

The authors declare that the research was conducted in the absence of any commercial or financial relationships that could be construed as a potential conflict of interest.

## Publisher’s Note

All claims expressed in this article are solely those of the authors and do not necessarily represent those of their affiliated organizations, or those of the publisher, the editors and the reviewers. Any product that may be evaluated in this article, or claim that may be made by its manufacturer, is not guaranteed or endorsed by the publisher.
